# NrtNet: An Unsupervised Method for 3D Non-Rigid Point Cloud Registration Based on Transformer

**DOI:** 10.3390/s22145128

**Published:** 2022-07-08

**Authors:** Xiaobo Hu, Dejun Zhang, Jinzhi Chen, Yiqi Wu, Yilin Chen

**Affiliations:** 1 School of Computer Science, China University of Geosciences, Wuhan 430074, China; huxiaobo@cug.edu.cn (X.H.); chenjinzhi@cug.edu.cn (J.C.); wuyq@cug.edu.cn (Y.W.); 2 Hubei Key Laboratory of Intelligent Robot (Wuhan Institute of Technology), Wuhan 430205, China; yilinchen@wit.edu.cn

**Keywords:** NrtNet, self-attentive, transformer, unsupervised, non-rigid point cloud, registration

## Abstract

Self-attention networks have revolutionized the field of natural language processing and have also made impressive progress in image analysis tasks. Corrnet3D proposes the idea of first obtaining the point cloud correspondence in point cloud registration. Inspired by these successes, we propose an unsupervised network for non-rigid point cloud registration, namely NrtNet, which is the first network using a transformer for unsupervised large deformation non-rigid point cloud registration. Specifically, NrtNet consists of a feature extraction module, a correspondence matrix generation module, and a reconstruction module. Feeding a pair of point clouds, our model first learns the point-by-point features and feeds them to the transformer-based correspondence matrix generation module, which utilizes the transformer to learn the correspondence probability between pairs of point sets, and then the correspondence probability matrix conducts normalization to obtain the correct point set corresponding matrix. We then permute the point clouds and learn the relative drift of the point pairs to reconstruct the point clouds for registration. Extensive experiments on synthetic and real datasets of non-rigid 3D shapes show that NrtNet outperforms state-of-the-art methods, including methods that use grids as input and methods that directly compute point drift.

## 1. Introduction

The 3D object has better flexibility, and with the continuous development of 3D sensing technology in recent years, the 3D point cloud has been widely used in various fields, such as virtual reality [1], autonomous driving [2], and augmented reality [3]. Since LIDAR scanned point clouds do not correspond with each other, this great inconveniences downstream tasks of point cloud classification [4,5], segmentation [6,7], registration [8,9], and reconstruction [10,11].

Non-rigid point cloud registration can be divided into similar registration [12,13] and affine registration [14,15]. Similar registration is mostly based on ICP to improve the registration of point clouds by changing the optimization objective function and increasing the correspondence, while affine registration ensures that the parallelism between the lines remains unchanged during the transformation process. Corrnet3D [16] proposes an alignment idea of finding the correspondence between the point clouds first, and then reconstructing the point clouds, which gives us inspiration. However, most of these methods require large-scale labeled data. Labeled data requires a lot of time and cost, which also promotes the development of unsupervised methods. In our work, we focus on unsupervised large deformation non-rigid point cloud registration, which means that only 3D point cloud data is required as input.

**Figure 1:**
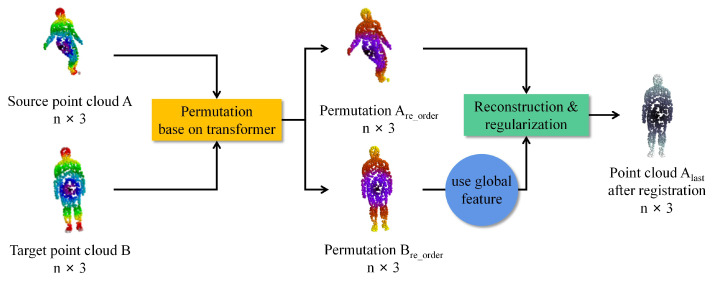
The idea of our proposed NrtNet. The point cloud is first rearranged using transformer, and then the permuted point cloud is reconstructed to achieve the registration.

Figure 1 illustrates our idea that if we can align two sets of point clouds *A* and *B*, the registration process between the point clouds becomes easy. We permutate the point clouds by a transformer because the transformer is better at handling natural language correspondences [17,18]. We design a reconstruction module to reconstruct $A_{re\underline{\;}order}\in R^{n\times 3}$ to *B*, which is more meaningful than reconstructing directly from *A* to *B*.

Based on the above idea, we propose an unsupervised transformer-based registration network (NrtNet) for large deformation non-rigid point clouds. We propose a transformer-based permutation process. Specifically, this permutation process uses the encoder and decoder of the transformer to generate a point set correspondence matrix, which represents the correspondence between the source point cloud *A* and target point cloud *B*. During the training process, the global features of the target point cloud and the permutation source point cloud $A_{re\underline{\;}order}$ are fed to the reconstruction module to obtain the reconstructed point cloud. The reconstruction module drives the learning of the correspondence matrix and the relative drift by optimizing the reconstruction error and additional regularization terms to achieve registration.

In general, our main contributions are:

- We propose a transformer-based point cloud correspondence learning framework for learning dense correspondences between point clouds, and we are the first to introduce a transformer into the field of non-rigid point cloud registration.
- Our network eliminates the reliance on ground truth and achieves unsupervised learning of non-rigid point cloud registration in an end-to-end manner, and has a better registration effect for different objects.
- Experiments demonstrate that NrtNet has significant advantages in non-rigid point cloud registration. In particular, it is superior to methods that directly compute the drift of coherent points between point clouds and methods that use a grid as input.

## 2. Related Work

In this section, we introduce the application of point clouds in deep learning, the study of non-rigid point cloud registration, and the development of transformer-based deep learning.

### 2.1. Deep Learning on Point Cloud

Compared with well-developed image-based deep learning methods, point cloud-based deep learning methods are more challenging and still in the developing stage due to the irregularity and disorder of point clouds. Three-dimensional data can be displayed in various forms, such as 2D multi-views, unstructured point clouds, voxelized volumes, etc. Voxelization methods convert 3D data into regular volume occupancy voxels, resulting in structured volumes that are well suited for 3D CNNs. Early point cloud tasks used end-to-end 3D convolutional networks [19,20,21]. Due to the sparse volume of 3D data and expensive 3D convolutions, voxelized representations are limited by resolution, and [22,23] effectively solve the voxelized resolution problem. Qi et al. [24] projected 3D data into multiple 2D views and used the popular 2D CNN to process it.

PointNet [25] learns features directly from the point cloud, maps the point cloud to higher dimensions before aggregation, and takes symmetry operations in higher dimensions. Mapping to higher dimensions generates redundant information, which can be captured by maximization operations to avoid geometric information loss. PointNet only uses MLP and max-pooling and does not have the ability to capture local structural defects, which PointNet++ [26] improves upon. DGCNN [27] designs an EdgeConv that can efficiently extract features of local shapes of point clouds while still maintaining alignment invariance. Later, researchers investigated the use of merged features to represent the overall features and pointwise features [26] or more sophisticated RNN-based methods to extract features [28,29]. MortonNet [30] extracts more effective features based on learning an ordered sequence of point clouds. FoldingNet [31] learns to deform predefined 2D regular meshes into 3D shapes, AtlasNet [32] and 3D-Coded [33] are also based on the deformation of their networks, and they use fixed template deformations to reconstruct the point cloud or mesh.

### 2.2. Non-Rigid Point Cloud Registration

The development of registration optimization algorithms has attracted the attention of many researchers, and these algorithms are used to refine geometric transformations during iterations. The Iterative Closest Point (ICP) algorithm [34] is a classic case in rigid registration. The ICP initializes the estimation of the rigidity function and then iteratively selects the corresponding point to revise the transformation. However, ICP is not able to handle non-rigid point cloud variations efficiently due to the influence of initial values. Non-rigid point cloud registration can be divided into parametric registration and non-parametric registration by target transformation. The TPS-RSM algorithm [35] in parametric registration estimates the parameters of the non-rigid transformation with the penalty of the second derivative.

For classical nonparametric methods, coherent point drift (CPD) [36] introduces a process of fitting a Gaussian mixed likelihood that aligns the source point set with the target point set. Ma et al. [37] proposed the importance of exploiting local and global structures in non-rigid point set registration. CPD-Net [38] uses deep neural networks to fit functions that can adapt to geometric transformations of varying complexity. DispVoxNets [39] converts point clouds to voxels for nonlinear deformation in a supervised manner. PR-Net [40] introduces point-set shape features that determine the correlation between the source and target point set to predict the transformation, allowing source and target point sets to be statistically aligned. CorrNet3D [27] uses a new efficient de-smoothing module to optimize the point set pairs with better results. Ma et al. [41] used a robust transformation estimation method based on streamwise regularization for non-rigid point set registration, and the spatial transformation between two point sets is estimated by iteratively recovering the point correspondence. However, all extant methods do not perform well for point cloud registration with large deformations, and most of them rely on ground truth. These methods also work poorly for data with non-corresponding point sets. Our method eliminates the reliance on ground truth and has better registration results for large deformations and non-corresponding data sets.

### 2.3. Deep Learning Based on Transformer

CNN is a standard network model in computer vision [42], with the introduction of AlexNet [43], CNN began to become the dominant network model. Transformer and Self-Attention models revolutionized natural language processing [44,45], and some studies used Self-attention and Transformer to replace some or all of the spatial convolutional layers in the popular ResNet [46]. The encoder-decoder design in Transformer has recently been applied to object detection and instance segmentation tasks [47], and ViT [48] directly applies transformer to non-overlapping medium-sized image blocks for image classification. AiR [49] is the first transformer-based image registration method. Point Transformer [50] is the first to introduce a transformer into the 3D point cloud domain, proposes a highly expressive point transform layer, and uses transformer to construct a high-performance point transform network for point cloud classification and dense prediction. Point Cloud Transformer [51] proposes a new transformer-based point cloud learning framework PCT, and uses implicit Laplace operators and normalized refinement to offset attention. Our method uses the transformer to derive correspondences between points to improve the effectiveness of the final registration.

## 3. Framework

### 3.1. Overview

**Figure 2:**
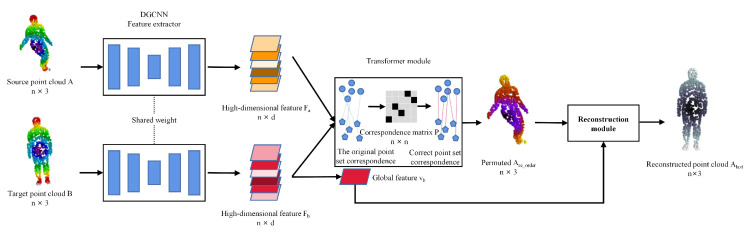
NrtNet is an unsupervised, end-to-end network for non-rigid point cloud registration. The source point cloud $A\in R^{n\times 3}$ and the target point cloud $B\in R^{n\times 3}$ are fed into the feature extraction module and the transformer module to generate the point set correspondence matrix $P\in R^{n\times n}$. Then, the permuted point cloud is fed into the reconstruction module to generate the exact same point cloud $A_{last}$ as *B*, which achieves the purpose of registration.

As shown in Figure 2, NrtNet is composed of three modules: the feature extraction module, the transformer module, and the point cloud reconstruction module. Firstly, in order to get the point cloud features, the source point cloud $A\in R^{n\times 3}$ and the target point cloud $B\in R^{n\times 3}$ are fed into the point cloud feature extraction module to generate point cloud features $F_{a}\in R^{n\times d}$ and $F_{b}\in R^{n\times d}$, where *d* is the feature dimension, the pointwise feature of the point cloud is obtained by setting d to the same dimension as the number of point clouds. After that, the pointwise features $F_{a}$ and $F_{b}$ are fed into the transformer module, which finds the correspondence between the source and target point clouds. The point set correspondence matrix $P\in R^{n\times n}$ is obtained to represent the point set correspondence, the parameter $p_{ij}=1$ of *P* represents the i-th point $a_{i}$ of the source point cloud and the j-th point $b_{j}$ of the target point cloud. The transformer module is composed of a transformer encoder and a transformer decoder. The source point cloud *A* is permuted using *P* to obtain $A_{re\underline{\;}order}\in R^{n\times 3}$. Finally, the global features of the target point cloud $V_{b}\in R^{d}$ and the permuted source point cloud $A_{re\underline{\;}order}$ are fed into the reconstruction module to obtain $A_{last}$, which is similar to the target point cloud *B*. The global features $V_{b}$ are aggregated from the pointwise features. As in most papers, we optimize our model parameters by minimizing the similarity between the reconstructed point cloud and the target point cloud. To better learn the correspondence between point sets, we regularize the point cloud correspondence matrix and then minimize it to obtain the optimal point set correspondence. We can express it as follows:

(1) $$M^{optimal}=argmin\left({∥B-A_{last}∥}_{F}^{2}+{∥A-B_{last}∥}_{F}^{2}+G\left(P\right)\right)\;,\;$$

where $A_{last}\in R^{n\times 3}$ and $B_{last}\in R^{n\times 3}$ are the point clouds after the registration, and ${∥.∥}_{F}$ represents the Frobenius parametric matrix. $G\left(P\right)$ is a regularization operation on the corresponding matrix.

### 3.2. Feature Extraction Module

For the feature extraction module, instead of using the traditional PointNet and PointNet++, we use a DGCNN with shared parameters to map points A and B to high-dimensional features. DGCNN uses edge convolution, EdgeConv to dynamically build graph structures on each layer of the network, using each point as a centroid to characterize its edge with each neighboring point feature, and then aggregates these features to obtain a new representation of that point. Firstly, DGCNN defines the edge feature representation as:

(2) $$e_{ij}=h\left(x_{i},x_{i}-x_{j}\right)$$

where *h* is that the edge convolution operation considers both the global information $x_{i}$, and the local neighborhood information $x_{i}-x_{j}$, and $x_{i}\in R^{1\times d}$ is the feature extracted by the i-th point fed into the edge convolution. Then, aggregating the edge features to obtain the feature $e_{ij}^{l+1}$ over the l-th layer is expressed as:

(3) $$e_{ij}^{l+1}=\chi_{x_{j}\in \Omega_{i}}h\left((x_{i},x_{i}-x_{j})\right)$$

where χ indicates that the aggregation operation consists of the MLP and max-pooling, Ω denotes the set of point-set pairs formed between the remaining points centered at point $x_{i}$ and the center point. After the multi-layer edge convolution, MLP and max-pooling operations, we can extract the pointwise features $F_{a}\in R^{n\times d}$ and $F_{b}\in R^{n\times d}$. The pointwise features are fed into a max-avg-pooling layer to get the global feature $V_{a}\in R^{d}$ and $V_{b}\in R^{d}$, which prepares for the later reconstruction.

### 3.3. Transformer Module

**Figure 3:**
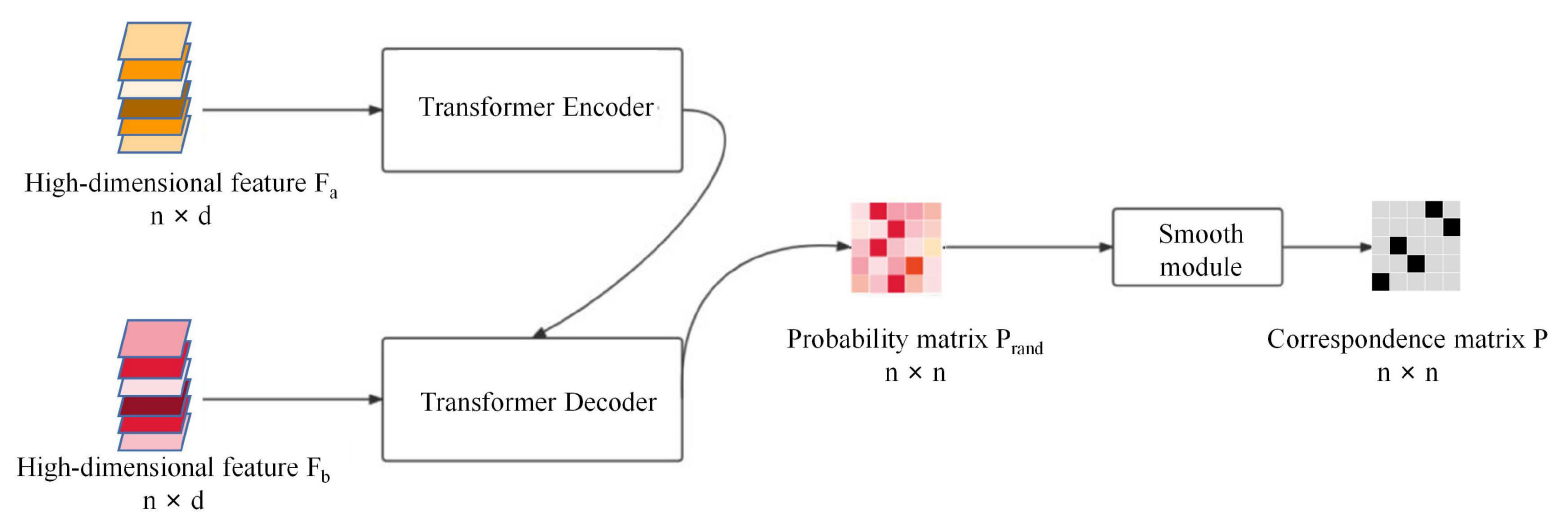
Transformer module. The probability matrix $P_{rand}$ can be obtained by feeding the high-dimensional features of the point cloud $F_{a}\in R^{n\times d}$ and $F_{b}\in R^{n\times d}$ into the transformer encoder and transformer decoder, respectively. Then, the probability matrix $P_{rand}$ is fed into the smooth module to obtain the inverted exact correspondence matrix *P*.

Because of the effectiveness of the transformer for word correspondence in NLP, we use the transformer to correspond to the point set. As shown in Figure 3, the transformer module consists of three parts: the transformer encoder, the transformer decoder, and a smooth module. The transformer module inputs the features of the source and target point clouds to learn the point set correspondence matrix $P\in R^{n\times n}$, which can explicitly represent the correspondence between any two points in *A* and *B*. The $p_{ij}=1$ in the matrix represents the i-th point $a_{i}$ of the source point cloud *A* and the j-th point $b_{j}$ of the target point cloud *B* are corresponding. This matrix is an inverted matrix. There are only two cases of correspondence between source and target point clouds, so this matrix should have only 0 or 1. The points in the source point cloud should correspond to the points in the target point cloud one by one, and each row and column of the matrix should have only one 1. The transformer module uses the transformer to find the similarity between point clouds, i.e., the probability matrix of the point clouds $P_{rand}$, which represents the probability of correspondence between the point clouds. Finally, a smoothing process is applied to this probability matrix to obtain an exact inverted binary matrix *P*.

**Figure 4:**
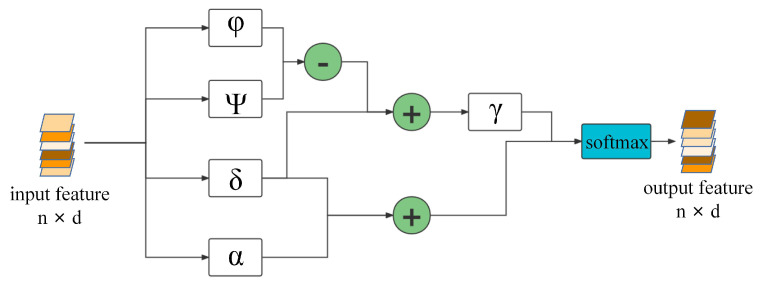
Transformer Encoder. φ, ψ is a linear layer, α is an mlp and they are all feature transform layers, δ is a linear layer which is a position encoder and γ is a mapping function.

The transformer encoder that references Point Transformer [50] is shown in Figure 4. The feature $f_{i}^{l}\in R^{l\times d}$ of the i-th point is fed into the standard scalar dot product attention layer. The standard scalar dot product attention layer is expressed as:

(4) $$y_{i}=\sum_{f_{i}^{a}\in F_{a}}^{}softmax\left(\gamma \left(\varphi {\left(f_{i}^{a}\right)}^{T}-\psi \left(f_{j}^{a}\right)\right)+\delta \right)\alpha \left(f_{j}^{a}\right)$$

where φ, ψ, α is the feature transform layer MLP, δ is a position encoder. γ is a mapping function. γ as a vector to represent the global features of the point cloud. The mapping function γ consists of an MLP, two linear layers, and a Relu activation function. Attention vectors are generated for later feature aggregation, feature transformation of φ minus ψ to obtain the vector relationship between them. Finally, the transformation features $y_{i}$ are obtained by a softmax regularization function.

Due to the disordered nature of point clouds and their irregular embedding in the entire vector space, self-supervision is performed using the position of the point cloud itself. The positional encoding δ is added to the transformed feature α. In this way, the transformation feature is expressed as:

(5) $$y_{i}=\sum_{f_{i}^{a}\in F_{a}\left(i\right)}^{}softmax\left(\gamma \left(\varphi {\left(f_{i}^{a}\right)}^{T}-\psi \left(f_{j}^{a}\right)\right)+\delta \right)\cdot \left(\alpha (f_{j}^{a})+\delta \right)\;,\;$$

where $F_{a}\left(i\right)\in F_{a}$ is the feature of *k* neighboring points around the sought point $f_{i}^{a}\in R^{l\times d}$. Self-attention is applied to each data point in the local domain. In 3D point cloud alignment, the 3D point cloud itself comes with position information, and the trainable parametric position encoder can be expressed as:

(6) $$\delta =\theta \left(p_{j}-p_{i}\right)\;,\;$$

where $p_{i}$ represent the i-th point, $p_{j}$ represents the j-th point around the i-th point, and θ has the same structure as γ. This position encoder has good effect enhancement for both attention generation and feature transformation.

**Figure 5:**
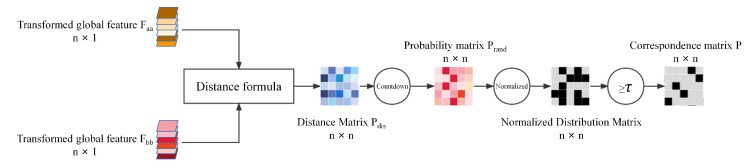
Transformer decoder and the smooth module. These two modules convert the transformed features obtained from the transformer encoder into an exact point set correspondence matrix.

As shown in Figure 5, the transformed features are fed into the transformer decoder and smoothing module to generate the point set correspondence matrix *P*. First, the global features need to be obtained by point-by-point features. The global feature can be expressed as:

(7) $$f_{aa}=\sum_{i=1}^{d}{f_{trans\underline{\;}i}^{a}}^{2}\;,\;f_{bb}=\sum_{j=1}^{d}{f_{trans\underline{\;}j}^{b}}^{2}$$

each transformed feature $f_{trans\underline{\;}i}^{a}$ is summed to obtain a one-dimensional global feature $f_{aa}$, so that we can find the global features $F_{aa}\in R^{n\times 1}$ and $F_{bb}\in R^{n\times 1}$ of the source and target point clouds. To obtain the probability matrix, we first obtain the distances of $F_{aa}$ and $F_{bb}$. The distance formula can be expressed as:

(8) $$P_{dis}={\left(F_{aa}\cdot I\right)}^{T}+F_{bb}\cdot I-2{\left(F_{aa}\cdot F_{bb}\right)}^{T}\;,\;$$

where *I* is a 1×n unit column vector. Equation (8) returns an n×n point set corresponding distance matrix. The larger the distance, the smaller the probability they correspond to. The probability matrix $P_{rand}$ corresponding to its point set is obtained by inverting the distance matrix. The probability matrix can be expressed as:

(9) $$P_{rand}=\frac{1}{P_{dis}}\;,\;$$

since there are only two cases for the correspondence of point sets, the probability matrix cannot effectively represent the correspondence between point sets. It can only represent the corresponding probability between point sets. We need to smooth this matrix, and we refer to Corrnet3D. Each row of the probability matrix should follow a normal distribution with mean $\mu_{i}$ and variance $\sigma_{i}$, i.e., $p_{rand\underline{\;}ij}∼N\left(\mu_{i},\sigma_{i}^{2}\right)$. In order to better filter the incorrect point set correspondence, we normalize this normal distribution $z_{ij}=\left(p_{rand\underline{\;}ij}-\mu_{i}\right)/\sigma_{i}$, $z_{ij}$ obeys the standard normal distribution $z_{ij}∼N\left(0,1\right)$. Finally, we select the corresponding point set according to the threshold τ. The number corresponding to the correct point set is $z_{num}$. For the points close to the middle, it should find a larger number of corresponding points, and $z_{num}$ should also obey the normal distribution. It obeys the three-sigma rule. The probability of the value in $\left[\mu_{Z_{num}}-3\sigma_{Z_{num}},\mu_{Z_{num}}+3\sigma_{Z_{num}}\right]$ is 0.9973, which is almost 1. The softmax operation on $z_{num}$ can be calculated to obtain the correct point set corresponding matrix *P*.

### 3.4. The Reconstruction Module

**Figure 6:**
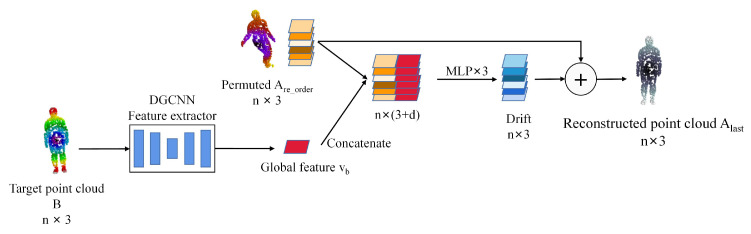
The reconstruction module concatenates the global features $v_{b}$ of the target point cloud to each permuted point cloud $A_{re\underline{\;}order}$, and feeds them into the MLP to reconstruct the point cloud $A_{last}$.

When the correct correspondence labels between point cloud *A* and *B* are given, the shape feature relationship between them can be learned well, and thus it is easy to learn the amount of drift between point sets. FoldingNet [31] and AtlasNet [32] reconstruct the global features by stitching point on top of the 2D grid. CPD-Net [38] learns point-to-point drift by concatenating point and global features. As shown in Figure 6, the reconstruction module based on point correspondence is proposed.

Point clouds *A* and *B* are permuted by the point set correspondence matrix *P*. *A* and *B* after permuting can be expressed as:

(10) $$A_{re\underline{\;}order}=P^{T}A\;,\;B_{re\underline{\;}order}=PB$$

The permuted source point cloud $A_{re\underline{\;}order}$ correspond to the point of *B* one by one, so that the large deformation registration can be learned, which CPD-Net cannot learn. The relative drifts between $A_{re\underline{\;}order}$ and *B* are learned by using the global features. As shown in Figure 6, $A_{re\underline{\;}order}$ and the global feature $V_{b}\in R^{d}$ are concatenated, and then the drift of each point is learned through three MLPs. The reconstructed point cloud $A_{last}$ is the source point cloud *A* plus the drift. The module is able to efficiently learn the drift between points for the purpose of registration. The reconstruction module learns a displacement field function to estimate the geometric transformations and is able to predict the geometric transformations of the alignment between positional objects.

### 3.5. Unsupervised Loss Function

The source point cloud $A_{last}$ should be similar to the target point cloud *B* after registration. The Euclidean distance loss between $B_{last}$ and *A* is added to the standard loss, which can better learn the relationship between *A* and *B*. According to the similarity of the source and target point clouds after deformation, the distance loss is expressed as:

(11) $$L_{dis}={∥B-A_{last}∥}^{2}+{∥A-B_{last}∥}^{2}$$

Since the points in *A* and *B* should be in one-to-one correspondence, their correspondence matrix should be an inverted matrix. The transpose of the inverted matrix and its own dot product should be infinitely close to the unit matrix. Based on this property, the matrix optimization loss formula is expressed as:

(12) $$L_{mat}={∥P^{T}P-I_{n}∥}^{2}\;,\;$$

where *P* is the correspondence matrix. $I_{n}$ is an n×n unit matrix.

There are similar local features between the target point cloud *B* and the source point cloud after the permutation $A_{re\underline{\;}order}$, and similarly the source point cloud *A* and the target point cloud after the permutation $B_{re\underline{\;}order}$ also have similar local features. Based on this property, the proximity similarity loss is expressed as:

(13) $$L_{pro}=\sum_{i=1}^{n}\left(\sum_{k\in \Omega_{i}^{a}}^{}\frac{{∥b_{re\underline{\;}i}-b_{re\underline{\;}j}∥}_{2}^{2}}{{∥a_{i}-a_{k}∥}_{2}^{2}}+\sum_{l\in \Omega_{i}^{b}}^{}\frac{{∥a_{re\underline{\;}i}-a_{re\underline{\;}j}∥}_{2}^{2}}{{∥b_{i}-b_{l}∥}_{2}^{2}}\right)\;,\;$$

where $\Omega_{i}^{a}$ represents the set of *k* indexes around the i-th point in *A*, $b_{re\underline{\;}i}\subset B_{re\underline{\;}order}$ and $a_{re\underline{\;}i}\subset A_{re\underline{\;}order}$ are the points after rearrangement.

Finally, we aggregate these losses and the final loss is expressed as:

(14) $$L=L_{dis}+\lambda L_{mat}+\eta L_{pro}\;,\;$$

where λ and η>0 are superparameters to regulate the balance between several losses.

## 4. Experiment

In this section, the experimental results of NrtNet’s non-rigid point cloud registration are presented. Details of the dataset and laboratory parameters used for training and testing are described in Section 4.1, and a brief introduction to the experimental evaluation method. In Section 4.2, a comparison of rigid point cloud registration results from different networks is discussed. In Section 4.3, the experimental results of non-rigid body methods in rigid registration are discussed. In Section 4.4, the registration results of NrtNet on small deformation datasets are presented. In Section 4.5, the effects of different losses on the experiments are compared. In Section 4.6, we show the registration effect of NrtNet on real scan data.

### 4.1. Experimental Setup

**Dataset**. We use the 200k sampled dataset from Surreal [33] as the unsupervised training datasets, and divide these 200k datasets into 100 random pairs for registration training. We used the 300 pairs dataset from Shrec [52] as the test dataset. We downsampled Shrec’s dataset to 1024 grids and took the grid vertices as input to keep the variables constant. In order to compare the robustness of different datasets, we used the dataset of Bednarik, J et al. [53] including small deformation datasets of paper, tshirt, sweater, and cloth to learn for different data to ensure the reliability of NrtNet.

**Evaluation**. We reviewed a large amount of information on whether CPD-Net [38], DispVoxNets [39], or other articles such as PR-Net [40] have most of the evaluations as direct comparison of CD loss or subjective comparisons of the experimental result plots after registration. Almost none of them had registration again by finding correspondence for point pairs like we do, so we refer to Corrnet3D’s [16] evaluation method to evaluate the goodness of the model based on whether the point set corresponds to each other or not. The point correspondence rate is expressed as:

(15) $$P_{rate}=\frac{1}{n}{∥P\circ P_{gt}∥}_{1}\;,\;$$

where ∘ is the Hadamard product and ${∥\cdot ∥}_{1}$ is the parametric matrix. $P_{gt}$ is the ground truth of the point set corresponding to the matrix. We set the percentage of correct correspondence under different tolerances to compare the pros and cons of the method. The point correspondence rate under different fault tolerance is expressed as:

(16) $$P_{corr}=\frac{r}{max\left\{{∥a_{i}-a_{j}∥}_{2}|\forall i,j\right\}}\;,\;$$

where *r* is the error tolerance radius.

**Experimental parameters and configuration**. We set the superparameter λ=0.1 and η=0.01. Our method was implemented in pytorch and our evaluation system was trained and tested on an NVIDIA GTX 1080 GPU. The learning-rate was 1 × 10${}^{-4}$, batchsize was two, and we trained 50 epochs on the large Surreal dataset [33] and 500 epochs on the small deformed dataset [53].

### 4.2. Experimental Evaluation of Non-Rigid Point Cloud Registration

**Figure 7:**
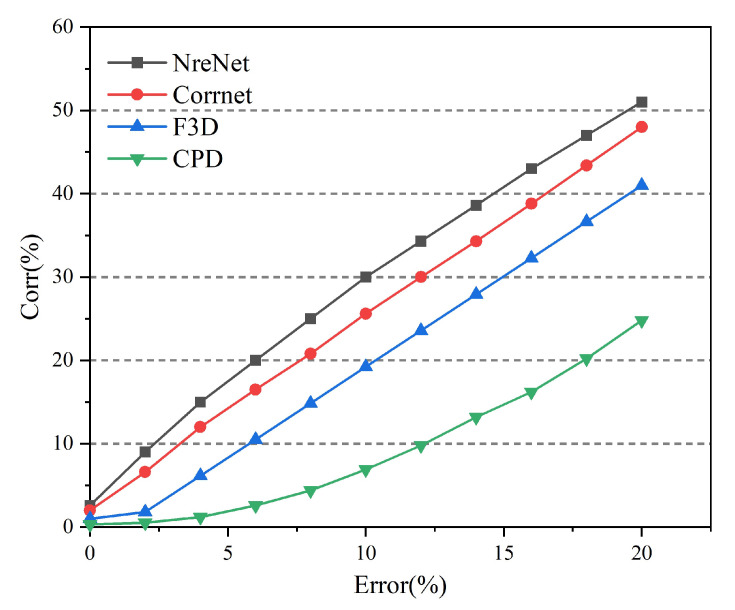
Quantitative comparison of point set correspondence rates for non-rigid registration under different methods.

NrtNet was compared with unsupervised FlowNet3D [54], unsupervised Corrnet3D [16], and unsupervised CPD-Net [38]. Figure 7 and Table 1 show a quantitative comparison of different methods, it can be seen that our method consistently outperforms other unsupervised methods. In particular, we have more significant performance advantages when comparing FlowNet3D and CPD-Net, and we also have some performance improvements when comparing Corrnet3D. The point set correspondence rate of CPD-Net is low, and the registration effect is poor for large deformation datasets. The point set correspondence rate of CPD-Net is low, and the registration effect is poor for large deformation datasets. Although FlowNet3D has a high correspondence rate, its registration effect is very dependent on the dataset, and some test datasets have a good registration effect, while some test datasets have a poor registration effect. Only NrtNet and the recently published Corrnet3D have better registration results. Because NrtNet uses a transformer that is better than Corrnet3D in point correspondence, it can still achieve better registration results for some datasets with larger deformations.

**Figure 8:**
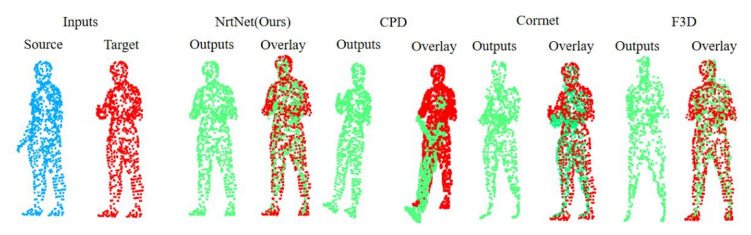
In a qualitative comparison between Nrtnet and other methods in a large deformed human pose, the experiments show the effectiveness of different methods for non-rigid point cloud registration.

Figure 8 shows the qualitative comparison results. NrtNet suffers less from unsupervised large-deformation non-rigid point cloud registration and can generate a point cloud with accurate correspondence. In contrast, CPD-Net and FlowNet3D are affected by large deformation, which makes their correspondence deviate and cannot achieve effective registration when the target point cloud varies greatly from the source point cloud. NrtNet learns the point set correspondence between the target and source point cloud, and thus can effectively make the registration effect better. Our network can further enhance the robustness to the degree of deformation by learning the specific type.

### 4.3. Experimental Evaluation of Rigid Point Cloud Registration

**Figure 9:**
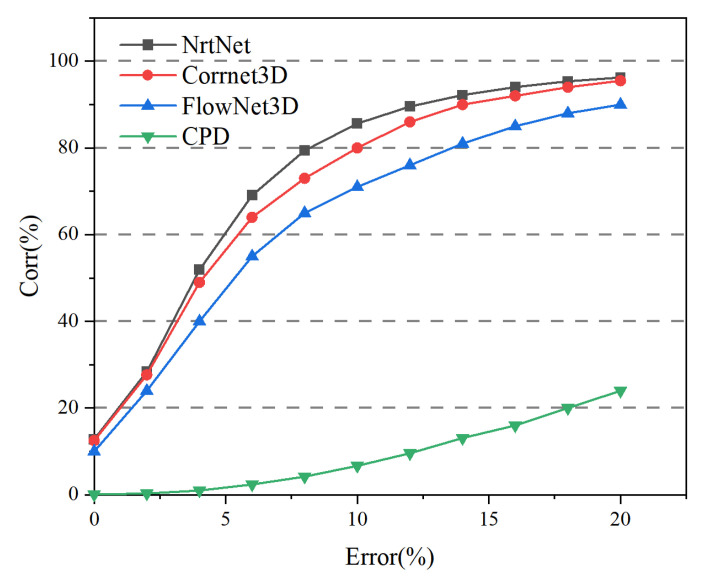
Quantitative comparison of point set correspondence rates for rigid registration under different methods.

We use the non-rigid registration method to register the rigid point cloud, and compare the effect of our method with FlowNet3D, Corrnet3D, and CPD-Net on the rigid point cloud registration. Figure 9 and Table 2 show our method and other methods compared with different fault tolerances. It can be seen from the table that our method has the best results under the same fault tolerance, Corrnet3D has a great improvement for FlowNet3D, and our method also has improvement for Corrnet3D. Compared with non-rigid point cloud registration, the unsupervised registration effect of CPD-Net in rigid point cloud registration has little improvement, while our method NrtNet has better registration effect and non-rigid point cloud registration in rigid point cloud registration.

### 4.4. Comparison between Different Datasets

**Figure 10:**
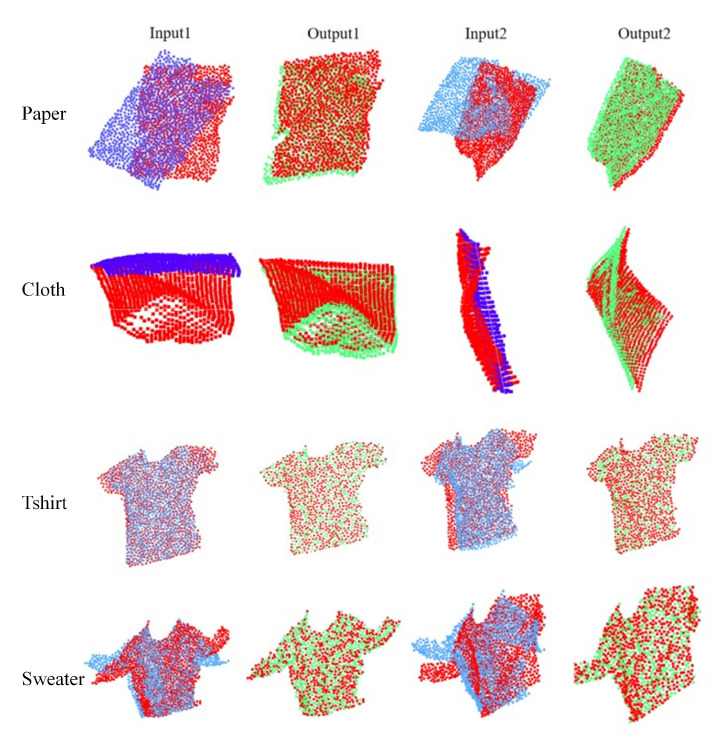
Registration performance of Nrtnet in small deformation dataset paper, cloth, sweater, and t-shirt.

Nrtnet was tested on the dataset of Bednarik, J et al. [53] for learning and registration to test the stability on different datasets. The dataset was divided into a training set and a test set in a ratio of 8:2. As shown in Figure 10, NrtNet has better registration for small deformation datasets, not only for learning the deformation part efficiently, but also for rigid transformations of deformed point clouds. NrtNet not only has a good registration effect on large deformation datasets, but also has good registration effects on small deformation datasets compared with existing methods. This makes the registration more efficient to first obtain the point set correspondence through the transformer.

### 4.5. Comparison of Different Losses

**Figure 11:**
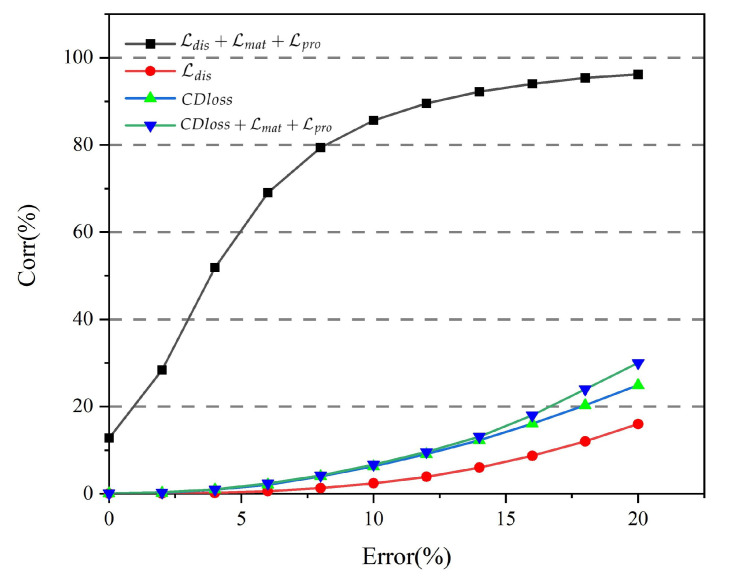
The point set correspondence rate under different losses. The experiments qualitatively compare the correspondence differences between NrtNet’s losses and ordinary losses.

In the experiment, we compared the difference between the Euclidean distance $L_{dis}$ and the CD Loss, and we also showed the improvement of the Euclidean distance and the cd distance by adding optimization losses $L_{mat}+L_{pro}$. Figure 11 and Table 3 show the point-set correspondence rate at different losses, and it can be seen that the loss of NrtNet achieves the best results with the same fault tolerance. It can be seen that the improvement of $L_{mat}+L_{pro}$ to $L_{dis}$ is very obvious by comparing $L_{dis}$ and $L_{dis}+L_{mat}+L_{pro}$. When CDloss and $L_{dis}$ are compared separately, CDloss has a certain improvement. When CDloss and $CDloss+L_{mat}+L_{pro}$ are compared separately, $L_{mat}+L_{pro}$ have little effect on CDloss, and the increase in correspondence rate is minimal. Experiments show that the loss of NrtNet can achieve the best experimental results.

### 4.6. Real Scan Data

**Figure 12:**
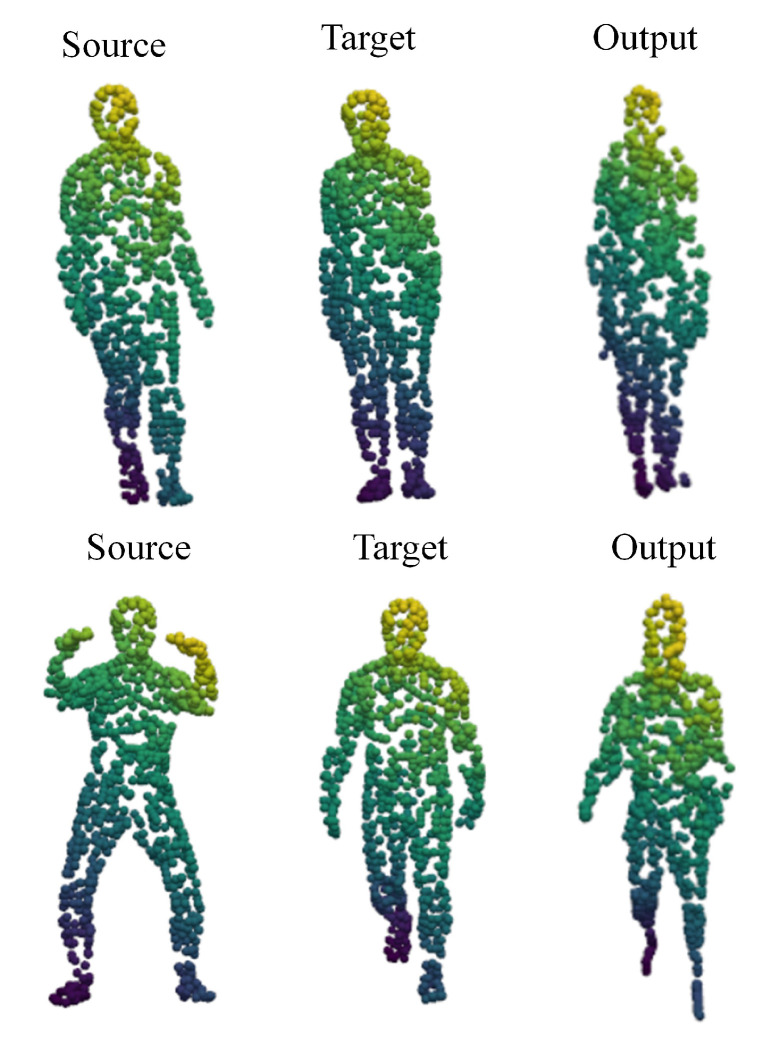
The registration effect of NrtNet on the real scan data.

This section shows the effect of NrtNet registration on real data. The experiments used Shrec’s human real scan dataset [52], and since the experiments were conducted without ground truth, it is hard to qualitatively evaluate the effects of the experiments. Figure 12 shows the final registration results of the experiments for a rational analysis of the results. NrtNet is able to effectively align the point cloud actions and shapes, and NrtNet is able to align any data without ground truth. As shown in Figure 12, The same color represents the correspondence of point sets, and NrtNet has better results for the correspondence of point set pairs. Although there are registration errors in some details, the experimental results are already much better than traditional non-rigid registration networks. The results are able to have better registration results for each movement.

## 5. Conclusions

We propose NrtNet, an unsupervised transformer-based registration architecture, which can learn the correspondence between pairs of large deformed point sets to effectively improve registration performance. NrtNet is much better than FlowNet3D in large deformation point cloud registration, and also significantly outperforms the state-of-the-art Corrnet3D. This shows that NrtNet can be used for most large deformation registration applications. We also show registration results on real scan data in the absence of ground truth, and still have good registration results. NrtNet has taken a long term step in large deformation non-rigid point cloud registration and eliminates the reliance on ground truth to conduct non-rigid point cloud registration.

In future work, NrtNet can be extended to voxels for non-rigid point cloud registration. Our correspondence may be inappropriate for the correspondence between points that are far apart. For this, we will sort the point cloud in future experiments and then use the transformer to do the point set correspondence, which corresponds to the word in NLP. Similarly, we believe that the registration effect can be improved to a certain extent after doing so. We believe that NrtNet can bring some help to other large scene point cloud registration, as well as human motion analysis and animal and plant growth analysis. Meanwhile, the model size of NrtNet can be further optimized to reduce training time.

## Figures and Tables

**Table 1:** Point set correspondence rates of different methods under different fault tolerance rates in non-rigid point cloud registration.

Method	0% Error Tolerance	10% Error Tolerance	20% Error Tolerance
CPD-Net	0.3311	6.8212	24.8963
FlowNet3D	1.2133	19.7614	41.3494
Corrnet3D	2.0494	25.68	48.8636
NrtNet	2.6889	30.0429	51.8758

**Table 2:** Point set correspondence rates of different methods under different fault tolerance rates in rigid point cloud registration.

Method	0% Error Tolerance	10% Error Tolerance	20% Error Tolerance
CPD-Net	0.1769	9.6191	24.286
FlowNet3D	10.8945	71.5576	90.368
Corrnet3D	12.4062	80.895	95.61
NrtNet	12.7793	85.6191	96.247

**Table 3:** Correspondence rates of point sets for different losses under different error tolerance rates.

Loss	0% Error Tolerance	10% Error Tolerance	20% Error Tolerance
$L_{dis}$	0.0214	2.4287	16.081
CDloss	0.0879	6.3662	24.9873
$CDloss+L_{mat}+L_{pro}$	0.1045	6.7686	30.7441
$L_{dis}+L_{mat}+L_{pro}$	12.7793	85.6191	96.247

## Data Availability

Our experiments employ open datasets in [33,52,53].

## References

[1] Apostolopoulos J.G., Chou P.A., Culbertson B., Kalker T., Trott M.D., Wee S. (2012). The road to immersive communication. Proc. IEEE.

[2] Raviteja T., Vedaraj I.R. (2020). An introduction of autonomous vehicles and a brief survey. J. Crit. Rev.

[3] Silva R., Oliveira J.C., Giraldi G.A. (2003). Introduction to augmented reality. Natl. Lab. Sci. Comput..

[4] Hackel T., Savinov N., Ladicky L., Wegner J.D., Schindler K., Pollefeys M. (2017). Semantic3d. net: A new large-scale point cloud classification benchmark. arXiv.

[5] Reisi A.R., Moradi M.H., Jamasb S. (2013). Classification and comparison of maximum power point tracking techniques for photovoltaic system: A review. Renew. Sustain. Energy Rev..

[6] Rabbani T., Van Den Heuvel F., Vosselmann G. (2006). Segmentation of point clouds using smoothness constraint. Int. Arch. Photogramm. Remote Sens. Spat. Inf. Sci..

[7] Brox T., Malik J. (2010). Object segmentation by long term analysis of point trajectories. Proceedings of the European Conference on Computer Vision.

[8] Myronenko A., Song X. (2010). Point set registration: Coherent point drift. IEEE Trans. Pattern Anal. Mach. Intell..

[9] Pomerleau F., Colas F., Siegwart R. (2015). A review of point cloud registration algorithms for mobile robotics. Found. Trends Robot..

[10] Tang P., Huber D., Akinci B., Lipman R., Lytle A. (2010). Automatic reconstruction of as-built building information models from laser-scanned point clouds: A review of related techniques. Autom. Constr..

[11] Vosselman G., Dijkman S. (2001). 3D building model reconstruction from point clouds and ground plans. Int. Arch. Photogramm. Remote Sens. Spat. Inf. Sci..

[12] Zha H., Ikuta M., Hasegawa T. (2000). Registration of range images with different scanning resolutions. Proceedings of the SMC 2000 Conference Proceedings, 2000 IEEE International Conference on Systems, Man and Cybernetics.’Cybernetics Evolving to Systems, Humans, Organizations, and Their Complex Interactions’.

[13] Zinßer T., Schmidt J., Niemann H. Point set registration with integrated scale estimation. Proceedings of the International Conference on Pattern Recognition and Image Processing.

[14] Amberg B., Romdhani S., Vetter T. (2007). Optimal step nonrigid ICP algorithms for surface registration. Proceedings of the 2007 IEEE Conference on Computer Vision and Pattern Recognition.

[15] Wang C., Shu Q., Yang Y., Yuan F. (2018). Point cloud registration in multidirectional affine transformation. IEEE Photonics J..

[16] Zeng Y., Qian Y., Zhu Z., Hou J., Yuan H., He Y. CorrNet3D: Unsupervised end-to-end learning of dense correspondence for 3D point clouds. Proceedings of the IEEE/CVF Conference on Computer Vision and Pattern Recognition.

[17] Vyas A., Katharopoulos A., Fleuret F. (2020). Fast transformers with clustered attention. Adv. Neural Inf. Process. Syst..

[18] Carion N., Massa F., Synnaeve G., Usunier N., Kirillov A., Zagoruyko S. (2020). End-to-end object detection with transformers. Proceedings of the European Conference on Computer Vision.

[19] Dai A., Chang A.X., Savva M., Halber M., Funkhouser T., Nießner M. Scannet: Richly-annotated 3d reconstructions of indoor scenes. Proceedings of the IEEE Conference on Computer Vision and Pattern Recognition.

[20] Huang J., You S. (2016). Point cloud labeling using 3d convolutional neural network. Proceedings of the 2016 23rd International Conference on Pattern Recognition (ICPR).

[21] Maturana D., Scherer S. (2015). Voxnet: A 3d convolutional neural network for real-time object recognition. Proceedings of the 2015 IEEE/RSJ International Conference on Intelligent Robots and Systems (IROS).

[22] Engelcke M., Rao D., Wang D.Z., Tong C.H., Posner I. (2017). Vote3deep: Fast object detection in 3d point clouds using efficient convolutional neural networks. Proceedings of the 2017 IEEE International Conference on Robotics and Automation (ICRA).

[23] Graham B. (2014). Spatially-sparse convolutional neural networks. arXiv.

[24] Qi C.R., Su H., Nießner M., Dai A., Yan M., Guibas L.J. Volumetric and multi-view cnns for object classification on 3d data. Proceedings of the IEEE Conference on Computer Vision and Pattern Recognition.

[25] Qi C.R., Su H., Mo K., Guibas L.J. Pointnet: Deep learning on point sets for 3d classification and segmentation. Proceedings of the IEEE Conference on Computer Vision and Pattern Recognition.

[26] Qi C.R., Yi L., Su H., Guibas L.J. (2017). Pointnet++: Deep hierarchical feature learning on point sets in a metric space. Adv. Neural Inf. Process. Syst..

[27] Phan A.V., Le Nguyen M., Nguyen Y.L.H., Bui L.T. (2018). Dgcnn: A convolutional neural network over large-scale labeled graphs. Neural Netw..

[28] Huang Q., Wang W., Neumann U. Recurrent slice networks for 3d segmentation of point clouds. Proceedings of the IEEE Conference on Computer Vision and Pattern Recognition, Salt Lake City.

[29] Ye X., Li J., Huang H., Du L., Zhang X. 3d recurrent neural networks with context fusion for point cloud semantic segmentation. Proceedings of the European Conference on Computer Vision (ECCV).

[30] Thabet A., Alwassel H., Ghanem B. (2019). Mortonnet: Self-supervised learning of local features in 3d point clouds. arXiv.

[31] Yang Y., Feng C., Shen Y., Tian D. Foldingnet: Point cloud auto-encoder via deep grid deformation. Proceedings of the IEEE Conference on Computer Vision and Pattern Recognition.

[32] Vakalopoulou M., Chassagnon G., Bus N., Marini R., Zacharaki E.I., Revel M.P., Paragios N. (2018). Atlasnet: Multi-atlas non-linear deep networks for medical image segmentation. Proceedings of the International Conference on Medical Image Computing and Computer-Assisted Intervention.

[33] Groueix T., Fisher M., Kim V.G., Russell B.C., Aubry M. 3d-coded: 3d correspondences by deep deformation. Proceedings of the European Conference on Computer Vision (ECCV).

[34] Besl P.J., McKay N.D. (1992). Method for registration of 3-D shapes. Proceedings of the Sensor Fusion IV: Control Paradigms and Data Structures.

[35] Chui H., Rangarajan A. (2000). A new algorithm for non-rigid point matching. Proceedings of the Proceedings IEEE Conference on Computer Vision and Pattern Recognition, CVPR 2000 (Cat. No. PR00662).

[36] Myronenko A., Song X., Carreira-Perpinan M. (2006). Non-rigid point set registration: Coherent point drift. Adv. Neural Inf. Process. Syst..

[37] Ma J., Zhao J., Yuille A.L. (2015). Non-rigid point set registration by preserving global and local structures. IEEE Trans. Image Process..

[38] Wang L., Li X., Chen J., Fang Y. (2019). Coherent point drift networks: Unsupervised learning of non-rigid point set registration. arXiv.

[39] Shimada S., Golyanik V., Tretschk E., Stricker D., Theobalt C. (2019). Dispvoxnets: Non-rigid point set alignment with supervised learning proxies. Proceedings of the 2019 International Conference on 3D Vision (3DV).

[40] Wang L., Chen J., Li X., Fang Y. (2019). Non-rigid point set registration networks. arXiv.

[41] Ma J., Wu J., Zhao J., Jiang J., Zhou H., Sheng Q.Z. (2018). Nonrigid point set registration with robust transformation learning under manifold regularization. IEEE Trans. Neural Netw. Learn. Syst..

[42] LeCun Y., Bottou L., Bengio Y., Haffner P. (1998). Gradient-based learning applied to document recognition. Proc. IEEE.

[43] Krizhevsky A., Sutskever I., Hinton G.E. (2012). Imagenet classification with deep convolutional neural networks. Adv. Neural Inf. Process. Syst..

[44] Vaswani A., Shazeer N., Parmar N., Uszkoreit J., Jones L., Gomez A.N., Kaiser Ł., Polosukhin I. (2017). Attention is all you need. Adv. Neural Inf. Process. Syst..

[45] Wu F., Fan A., Baevski A., Dauphin Y.N., Auli M. (2019). Pay less attention with lightweight and dynamic convolutions. arXiv.

[46] Tchapmi L., Choy C., Armeni I., Gwak J., Savarese S. (2017). Segcloud: Semantic segmentation of 3d point clouds. Proceedings of the 2017 International Conference on 3D Vision (3DV).

[47] Dovrat O., Lang I., Avidan S. Learning to sample. Proceedings of the IEEE/CVF Conference on Computer Vision and Pattern Recognition.

[48] Dosovitskiy A., Beyer L., Kolesnikov A., Weissenborn D., Zhai X., Unterthiner T., Dehghani M., Minderer M., Heigold G., Gelly S. (2020). An image is worth 16x16 words: Transformers for image recognition at scale. arXiv.

[49] Wang Z., Delingette H. (2021). Attention for Image Registration (AiR): An unsupervised Transformer approach. arXiv.

[50] Zhao H., Jiang L., Jia J., Torr P.H., Koltun V. Point transformer. Proceedings of the IEEE/CVF International Conference on Computer Vision.

[51] Guo M.H., Cai J.X., Liu Z.N., Mu T.J., Martin R.R., Hu S.M. (2021). Pct: Point cloud transformer. Comput. Vis. Media.

[52] Donati N., Sharma A., Ovsjanikov M. Deep geometric functional maps: Robust feature learning for shape correspondence. Proceedings of the IEEE/CVF Conference on Computer Vision and Pattern Recognition.

[53] Bednarik J., Fua P., Salzmann M. (2018). Learning to reconstruct texture-less deformable surfaces from a single view. Proceedings of the 2018 International Conference on 3D Vision (3DV).

[54] Liu X., Qi C.R., Guibas L.J. Flownet3d: Learning scene flow in 3d point clouds. Proceedings of the IEEE/CVF Conference on Computer Vision and Pattern Recognition.

